# Metagenomics reveals cryptic circulation of zoonotic viruses in Nigeria

**DOI:** 10.21203/rs.3.rs-7630852/v1

**Published:** 2025-09-23

**Authors:** Anise Happi, Ayotunde Sijuwola, Ifeanyi F. Omah, Olusola Ogunsanya, Femi Saibu, Akeemat Ayinla, Oluwatobi Adedokun, John Fadele, Cecilia Nwofoke, Ademola Adelabu, Ebenezer Ogundana, Omolola Lawal, Obineche Elias, Emmanuel Okokoh, Rachel Colquhoun, Olivia Achonduh-Atijegbe, Henshaw Nta, Ali Onimajesin, Fawaz Momoh, Almudena Mari-Saez, David Redding, Kris Murray, Johanna Hanefeld, Abdul Karim Sesay, Andrew Rambaut, Christian Happi

**Affiliations:** African Center of Excellence for Genomics of Infectious Diseases, Redeemer’s University, Ede, Osun State, Nigeria; Institute of Genomics and Global Health (IGH), Redeemer's University; Institute of Ecology and Evolution, University of Edinburgh, The King's Buildings, Edinburgh EH9 3FL, UK.; Institute of Genomics and Global Health; Institute of Genomics and Global Health (IGH), Redeemer's University; Institute of Genomics and Global Health (IGH), Redeemer's University; Institute of Genomics and Global Health (IGH), Redeemer's University; Institute of Genomics and Global Health (IGH), Redeemer's University; Alex Ekwueme Federal University Teaching Hospital; Institute of Genomics and Global Health (IGH), Redeemer's University; Institute of Genomics and Global Health (IGH), Redeemer's University; Institute of Genomics and Global Health (IGH), Redeemer's University; Institute of Genomics and Global Health (IGH), Redeemer's University; Institute of Genomics and Global Health (IGH), Redeemer's University; University of Edinburgh; Institute of Genomics and Global Health (IGH), Redeemer's University; Institute of Genomics and Global Health (IGH), Redeemer's University; Institute of Genomics and Global Health (IGH), Redeemer's University; Institute of Genomics and Global Health (IGH), Redeemer's University; L’Institut Bouisson Bertrand, 5 rue Ecole de Médecine; Natural History Museum; Medical Research Council Unit The Gambia at London School of Hygiene and Tropical Medicine (LSHTM); Robert Koch Institute; Medical Research Council Unit The Gambia at London School of Hygiene and Tropical Medicine (LSHTM); University of Edinburgh; Redeemer's University

## Abstract

Zoonotic spillover events pose an ongoing threat to global health, with historic and recent viral diseases of international concern emerging from animal reservoirs 1–6. In Nigeria, limited surveillance of animal hosts at the human and animal interface continues to hinder our understanding of viruses that are cryptically circulating in animals near human dwellings with potential for consequential spillover events. We performed unbiased metagenomic next-generation sequencing (mNGS) on tissue and swab samples collected from 240 individual animals across 11 taxa (rodents, shrews, bats, goats, sheep, pigs, dogs, cats, chickens, cattle egrets, and lizards) in two Lassa-affected Nigerian states (Ondo and Ebonyi). Host-depleted sequencing reads were assembled into contigs, taxonomically classified, and subjected to phylogenetic analyses to characterize viral diversity, host associations, and evidence of cross-species transmission. Across all samples, we identified 214 distinct viral taxa spanning 33 families, of which 41% (n = 83) represent novel species by ICTV criteria. Positive-sense RNA viruses dominated (Coronaviridae, Picornaviridae, Astroviridae), followed by negative-sense RNA, single- and double-stranded DNA, and double-stranded RNA viruses. Notably, human-associated enteroviruses—including Hepatitis A virus (genotype 1b), echoviruses, coxsackieviruses, and noroviruses—were detected in goats, pigs, dogs, and chickens, indicating cryptic circulation of human pathogens in peridomestic and domesticated animals. Phylogenetic reconstructions revealed multiple cross-species viral sharing events, particularly among rodents, goats, sheep, and pigs, and extensive recombination within Nigerian Betacoronavirus 1 lineages. Interestingly we found a putative novel avian like coronavirus in rodents, goats and sheep. Ecological modelling demonstrated that host species identity, sample type, and sampling effort were primary drivers of viral richness and abundance, and that higher overall viral diversity strongly predicted cross-species transmission potential. Our integrated mNGS approach uncovered a rich and dynamic virome within animals inhabiting human-dominated environments in Nigeria, including undetected circulation of human enteric viruses. These findings underscore the importance of broad-taxonomic, real-time surveillance at human–animal interfaces to inform early-warning systems and pandemic preparedness, particularly in low-resource settings.

## Introduction

Seven of the eight viral diseases declared Public Health Emergencies of International Concern by the World Health Organization originated from zoonotic events^1–6^. Current outbreak responses remain largely reactive, highlighting the critical, but often difficult need to identify and interrupt these spillover events before they seed human epidemics, particularly in low-resource where long-term surveillance and response capacity are limited. Although viruses routinely cross species barriers, only a small fraction of these stochastic events progress to sustained human transmissions^7–9^. The ones that do, overcome a suite of ecological, epidemiological, immunological, and behavioral barriers which are often complex and dynamic^9–11^. The fundamental requirement of spillovers, however, is the presence of a pathogen within a non-human organism, so without prompt detection and characterization of emerging pathogens in reservoir or intermediate hosts, isolated spillover events can morph into large-scale epidemics, or pandemic which is often facilitated by population growth and interconnected travel networks^12–14^. Ebolavirus^11^ Influenza^15^, MERS-CoV^16^, SARS-CoV-2^6^ and recent Mpox outbreak^17,18,19^ have exemplified these scenarios well.

Metagenomic next-generation sequencing (mNGS) has revolutionized infectious disease surveillance and holds a promise for real-time monitoring of community viromes and early identification of emerging pathogens^9,10,20^. Despite this potential, most publicly available metagenomic data remain biased toward humans and economically or epidemiologically important wildlife hosts with limited data from low resource settings^9,21,22^. Recent studies have expanded sampling efforts to encompass invertebrates and marine animal taxa, broadening our understanding of viral diversity and evolution^22–24^. However, the extensive diversity of animal viruses identified through metagenomics complicates predictions about which viruses may eventually emerge in humans^20,21,25^. Long-term phylogenetic analyses of virus host relationship offer limited predictive power for short-term zoonotic risks^2^, underscoring the necessity of targeted surveillance at critical human–animal interfaces, particularly in resource-limited regions where humans, domestic animals, peridomestic wildlife, and vectors coexist densely^16,20,25,26^. These interfaces represent critical hotspots for spillover events^16,18,26^.

Small mammals, particularly rodents and bats, are recognized reservoirs for high-consequence viruses, including Ebola virus, Lassa virus, and coronaviruses^9,27,28^. Recent RT-PCR studies in Nigeria have identified Lassa virus RNA in unconventional hosts such as goats, pigs, lizards (red-headed agama), and dogs, highlighting critical surveillance gaps^29^. Hence the utility of integrated metagenomic surveillance of humans and animals to inform comprehensive control strategies of both endemic and emerging viruses with pandemic potential through an ecological framework^9,10,30^.

Nigeria lies within regions identified as having substantial numbers of undocumented viruses and zoonoses, marking it as a priority area for surveillance^2,29^. Given the prevalence of subsistence farming and the free roaming of animals within human settlements, both humans and their domesticated animals coexist across urban and rural areas^2,31^. This shared ecosystem fosters direct and indirect interspecies contact, creating a web of interactions at these interfaces that elevates the risk of spillover events. To address this critical gap, we applied mNGS to 240 individual animals representing 11 distinct taxa from two Nigerian states previously impacted by Lassa outbreaks. Our analysis uncovered human viruses in these animals that are of epidemic potential. Understanding the diversity, evolutionary dynamics, and zoonotic potential of viruses at this complex human–animal interface, is critical for risk assessments, early warning systems, and pandemic preparedness strategies.

## Results

### Multiple host taxa sampled across different ecological settings

**Figure 1:**
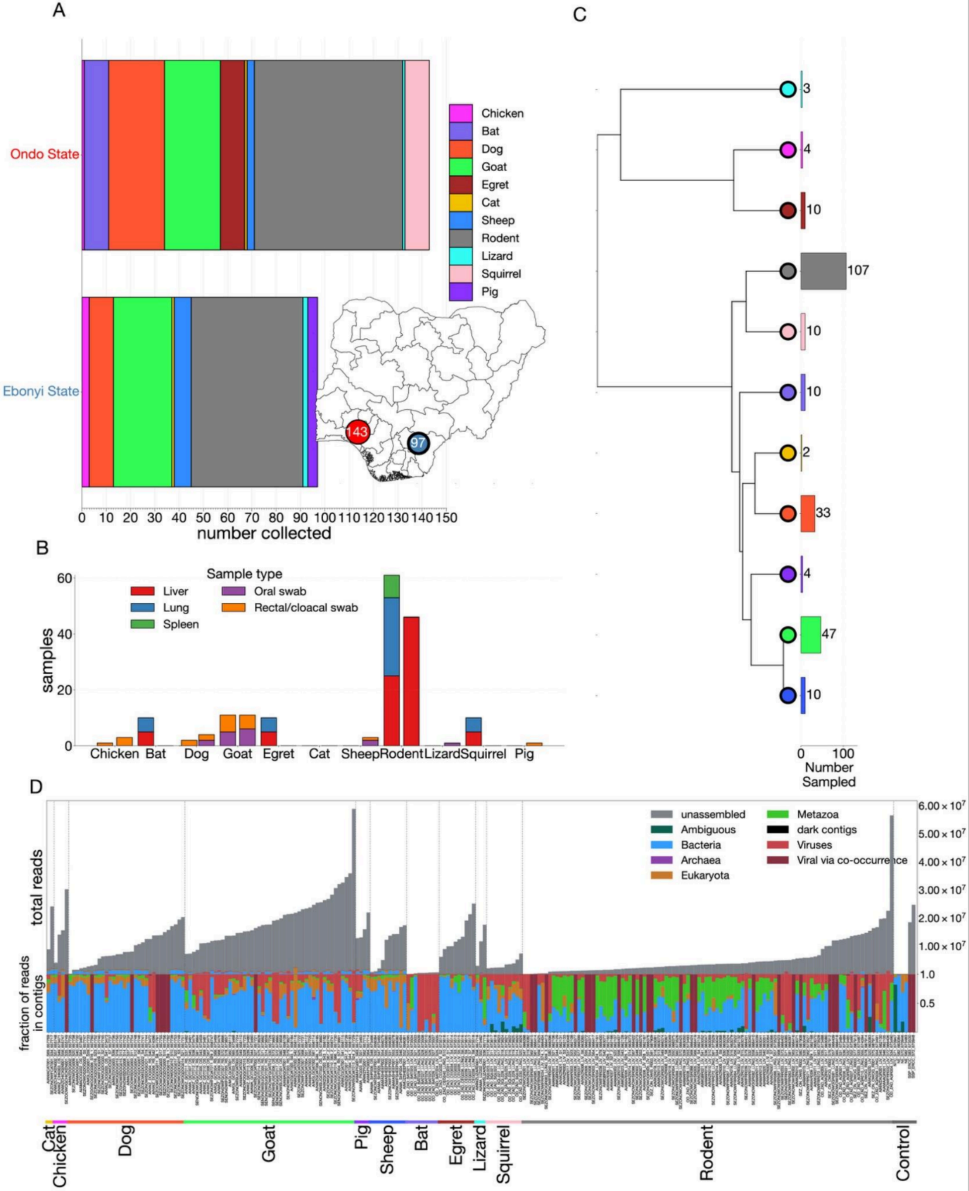
Sampling, host context, and metagenomic composition. **a**, Counts of host species sampled in Ondo (top) and Ebonyi (bottom). Colours denote host taxa. Inset: map showing the locations of both states in Nigeria. **b,** Specimen types collected per individual by state and host: tissues (liver, lung, spleen) and swabs (oral, rectal/cloacal). **c**, Phylogeny of sampled hosts derived from TimeTree; tip colours match panel A. Bars adjacent to tips indicate the number of viruses detected per host. **d,** Read and contig composition per sample. Upper panel: stacked bars showing total reads per sample. Lower panel: proportional distribution of assembled contigs across categories—Bacteria, Archaea, Eukaryota (Metazoa), Viruses (reference-aligned), Viruses (co-occurrence; see Methods), Ambiguous (LCA at root or “cellular organisms”), Dark (assembled but taxonomically unassigned), and Unassembled reads (did not form contigs). Viral reads were identified by alignment to reference databases and by viral co-occurrence analysis (Methods).

We sampled 240 animals from 11 host taxa—including domestic, peri-domestic, and wild species—from Ondo (n=143) and Ebonyi (n=97) States (Fig. 1A). Wild animals were identified to at least the family level using morphological characters and mitochondrial cytochrome oxidase (COI). The collection sites differed environmentally: Ondo had denser forest and higher rainfall, whereas Ebonyi had sparser tree cover, lower rainfall, and fewer wild animals. We sampled greater host diversity in Ondo (10 taxa) compared to Ebonyi (8 taxa). Small mammals sampled included rodents of the genus (*Mastomys, Cricetomys, Rattus, Mus*), squirrels (*family Sciuridae*), and shrews (*Suncus; family Soricidae*); other hosts included bats (*Eidolon helvum*), goats, sheep, dogs, pigs, cats, birds—cattle egrets (*Bubulcus ibis*) and chickens—and reptiles (the red headed agamid lizards; family *Agamidae*) (Fig. 1B–C). Rodents (*Mastomys, Rattus, Mus*) were the most common host sampled; cattle egrets, bats, and squirrels were unique to Ondo, whereas pigs were sampled only in Ebonyi (Fig. 1A, C). Liver, lung, and spleen tissues were collected from rodents, bats, and cattle egrets, while oral and rectal/cloacal swabs were taken from other hosts (Fig. 1B). Our sampling was done to reflect the multiple animal taxa that are common around human habitats in Ondo and Ebonyi states and wild species that frequently interact with domestic animals at the human–wildlife interface.

To characterize host viromes, we sequenced tissues and swabs from sampled animals (Fig. 1B). Across all libraries, metagenomic NGS yielded 7.3 billion reads, which assembled into 4.1 million contigs. Assembled contigs were taxonomically annotated against NCBI nr/nt database to profile the virome and broader microbiota per host (Fig. 1D; Supplementary Figs. S1–S2). For classification of viruses, we prioritised complete/near-complete RNA viral genomes and curated RNA-dependent RNA polymerase (RdRp) contigs, supported by co-occurring contigs linked to each RdRp while DNA polymerase and replicase were used for DNA viruses. Bacterial sequences dominated the non-host reads, followed by retroviruses and Viridiplantae (Figs. S2); these taxa are beyond the scope of this study and will not be discussed further. Host-derived (Chordata) contigs supported host identification to at least family level.

**Figure 2:**
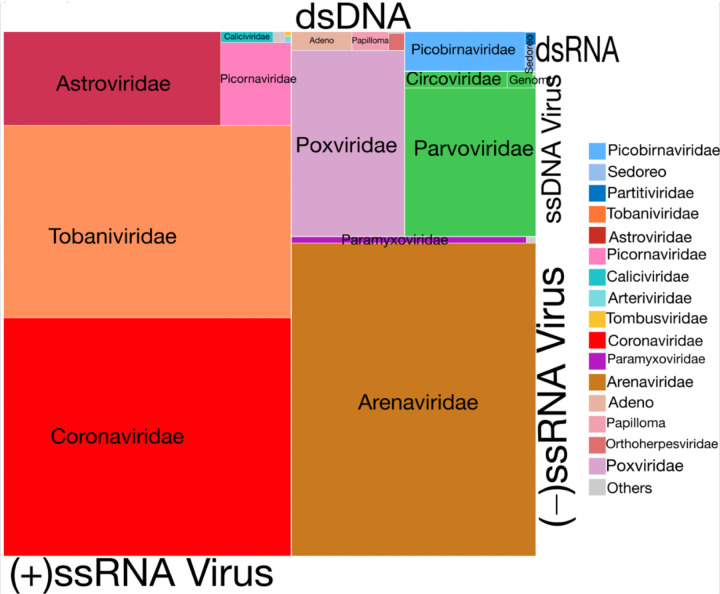
A family level resolution breakdown of microbial signatures across 240 samples. This is visualised as a treemap where each family of viruses identified are represented by squares shaded by taxon identity (see legend) and scaled in size according to read counts (supplementary figure 2 for a higher resolution). (+)ssRNA and (-)ssRNA dominate the viromes community

**Figure 3:**
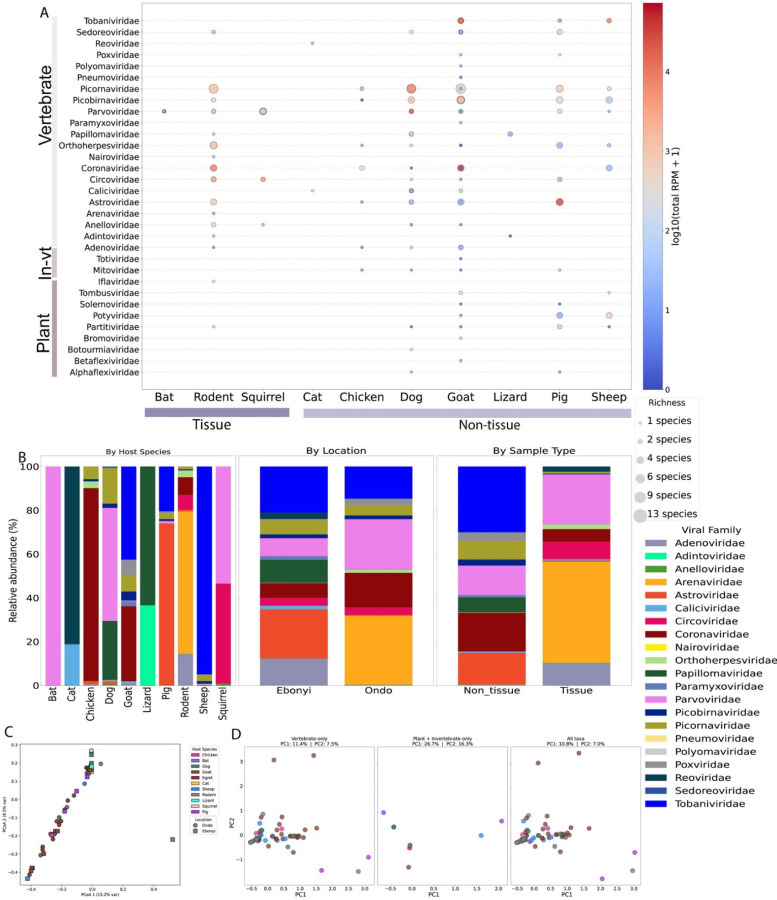
Viral diversity and distribution across sampled animal hosts. **a,** Bubble plot depicting viral families identified across host species categorized by vertebrate, invertebrate (In-vt), and plant origin. Bubble size indicates species richness, while colour intensity represents log-transformed reads per million (RPM). **b,** Bar plots illustrate the relative abundance of viral families identified by host species, sampling location (Ebonyi and Ondo states), and sample type (tissue vs. non-tissue). **c**, Principal coordinate analysis (PCoA) based on Bray-Curtis dissimilarities, showing clustering patterns of viral communities in samples from Ebonyi and Ondo states. **d,** Principal component analysis (PCA) demonstrates differentiation among viral communities, separately analysed for vertebrate-only, plant plus invertebrate-only, and combined taxa datasets. Colours indicate host species; shapes represent sampling locations.

Using phylogenetic analyses of hallmark genes, RNA-dependent RNA polymerase (RdRp) for RNA viruses and DNA polymerase/replicase for DNA viruses, we identified 214 viral species from 33 families across hosts (21 RNA families, 12 DNA families), spanning vertebrate-, invertebrate-, and plant-associated viruses, with a significant gradient in representation across these categories (vertebrate > invertebrate > plant; p < 0.05; Figs. 2–3A; Supplementary Fig. S4; Supplementary Table S1). Of these, 121 were closely related or identical to previously reported human or animal viruses (Oguzie *et al*., 2023; Shi *et al*., 2016, 2018) with amino acid identity > 90%. The remaining 83 species, ~39% of detections, met the ICTV (2022) species demarcation criteria and were classified as novel (Supplementary Table S2).

The diversity and relative abundance of viruses varied across host taxa and sample type (Fig. 3A–C; Supplementary Fig. S4). Positive-sense single-stranded RNA viruses dominated, accounting for ~5.4 million viral reads. A treemap (Fig. 2) and heatmap (Fig. 3A) highlight the major, predominantly vertebrate-associated viral families detected across hosts and sample types (Fig. 3B). *Picornaviridae, Coronaviridae*, and *Astroviridae* were widespread, occurring in six of eleven host taxa (e.g., chicken, goat, pig, sheep, dog, and *Mastomys*), and were significantly more frequent in non-tissue (swab) than tissue samples (*Picornaviridae*: 11/49 tissue vs 44/49 non-tissue, χ^2^=42.43, P=7.32×10^−11^; *Coronaviridae*: 6/14 vs 12/14, P=0.046; *Astroviridae*: 5/26 vs 24/26, P=1.23×10^−7^; Fig. 3A–B). *Tobaniviridae* and *Caliciviridae* were detected exclusively in oral/rectal swabs (P<0.05) at high relative abundance in sheep, goats, pigs, and cats (Fig. 3B). Worthy of note is that many of the virus families detected are of veterinary and zoonotic concern.

Negative-sense single-stranded RNA viruses comprised the second-largest viral category, with approximately 2.8 million viral reads. As shown in Figures 2 and 3A, prominent families within this category were highlighted in the treemap. These viruses were more host-restricted and less often detected compared to positive-sense RNA viruses. *Arenaviridae*, the most abundant family and known to have caused major outbreaks of haemorrhagic fever in Nigeria^29,32,33^, were exclusively found in seven liver and one lung samples from *Mastomys coucha* which is a natural host of Lassa virus sampled in Ondo State, with no presence in the samples from Ebonyi State (Figure 3B). *Paramyxoviridae*, known for their significant impact on livestock, were frequently identified in goat samples from Ebonyi State (Figure 3B). *Nairoviridae*, major causes of hemorrhagic diseases^34^, were primarily detected in the Shrew sample (genus: *Suncus sp*) (Figure 3A, B, supplementary table 4). Despite lower abundance than Positive-sense single-stranded, these families include highly pathogenic taxa.

Although the single-stranded RNA viruses dominated the virome abundance, double-stranded and single-stranded DNA viruses, which accounted for the third and fourth most abundant category, 0.8 and 0.7 million viral reads, respectively, have a wider host range (Kruskal–Wallis p = 0.0306). Figure 3A–B highlights representative dsDNA virus families including *Poxviridae, Adenoviridae*, and *Papillomaviridae*. The dsRNA viruses were the fifth and least abundant, although we identified only *Picorbirnaviridae* as the family in this category, but they appear as the viral family widely distributed across six of eleven hosts and sample types, although more in non-tissue than tissue samples (p<0.05), Figure 3B. This virus has also been widely reported in several metagenomic studies in Lizards, goats, and small mammals^35–37^. This highlights the ubiquity of these viruses and their potential zoonotic risk. Collectively, some of the viruses described in this study have been previously reported to cause a major outbreak of public health and veterinary concern^29^.

Community structure was explained primarily by host (PERMANOVA pseudo-F=2.04, P=0.003; 999 permutations), with a borderline effect of sample type on richness (χ^2^=3.76, df=1, P=0.053) and no effect of location (χ^2^=0.08, df=1, P=0.78) (Figs. 2C–D). Principal coordinates analysis (Bray–Curtis) showed strong host-associated clustering and extensive overlap between Ondo and Ebonyi (PC1 = 15.3% variance; PC2 = 9.5%; Fig. 3C). Subsetting to vertebrate-associated viruses preserved host structure (PC1 = 11.4%, PC2 = 7.5%), whereas plant/invertebrate-associated viruses yielded diffuse ordinations (PC1 = 26.7%, PC2 = 16.3%); combining all taxa dampened the vertebrate signal (PC1 = 10.8%, PC2 = 7.0%) (Fig. 3D). These patterns align with prior work indicating host traits and sample type as key drivers of virome variation^37^. The swab-dominant signal for several families suggests per-animal paired tissue–swab sampling will better resolve tissue tropism and potential cross-species transmission.

Phylogenetics analysis reveals the co-circulation of human and animal viruses.

**Figure 4:**
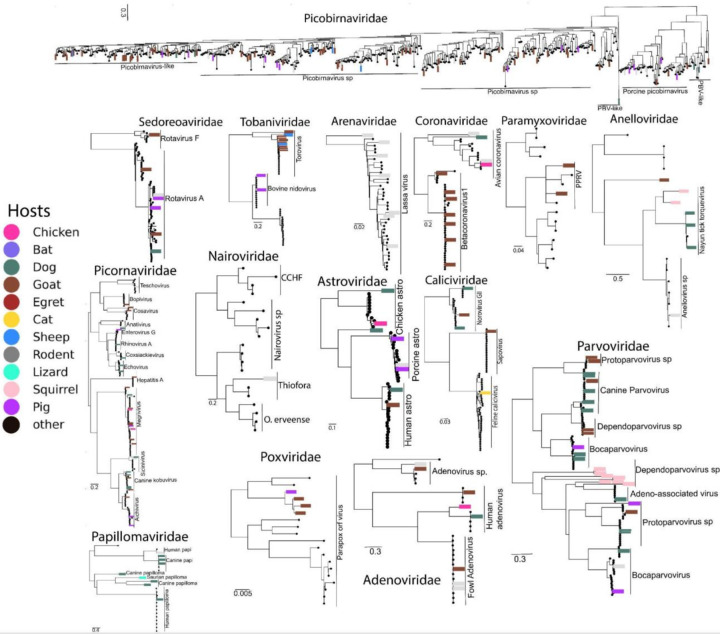
Maximum-likelihood amino-acid phylogenies of vertebrate-associated RNA and DNA viruses identified in this study. All trees are midpoint-rooted. PBVs *Piconabirnavirus, CCHF*, Crimean–Congo haemorrhagic fever virus; *PPRV*, peste des petits ruminants virus.

To place the detected viruses in evolutionary context, we inferred phylogenies from hallmark genes (RdRp; DNA polymerase/replicase). Most key nodes are well supported (bootstrap 70–99%), except where indicated (Fig. 4). We identified a novel rodent-associated gammacoronavirus that shared approximately <80% (aa) identity with the avian infectious bronchitis virus (IBV) RdRp. We propose the provisional name “Rodent coronavirus“ (RCoV) for this lineage. This virus clustered tightly with IBV strains isolated from chickens in Guinea. Based on our co-occurrence analysis we recovered other segments of this virus, the nucleocapsid, the spike region with 67% and 65% amino acid identity to avian coronavirus respectively. The phylogeny of the spike (S) gene and nucleocapsid clustered with other avian strains (Supplementary Figure 5). Other short viral RdRp contigs exhibiting >90% aa identity to avian coronavirus viruses were detected in samples from dogs, chickens, and goats, clustering together with avian coronaviruses. However, their placement within the phylogeny lacked robust bootstrap support (<50%; Figure 3, Supplementary Figure 5). While it is expected to detect avian coronavirus in birds, detecting this virus in unconventional hosts, rodents, dogs, and goats is indicative of several of the possible cross-species transmission that go undetected in our complex ecosystem^9,10^. The sequences from rodents, dogs, and goats were from Ondo State, indicating potential proximity-driven viral sharing through shared food sources or habitat overlap. The detection in Ebonyi, over 400 km away from Ondo State, further suggests the possible endemicity of these viruses among animal populations in Nigeria.

**Figure 5:**
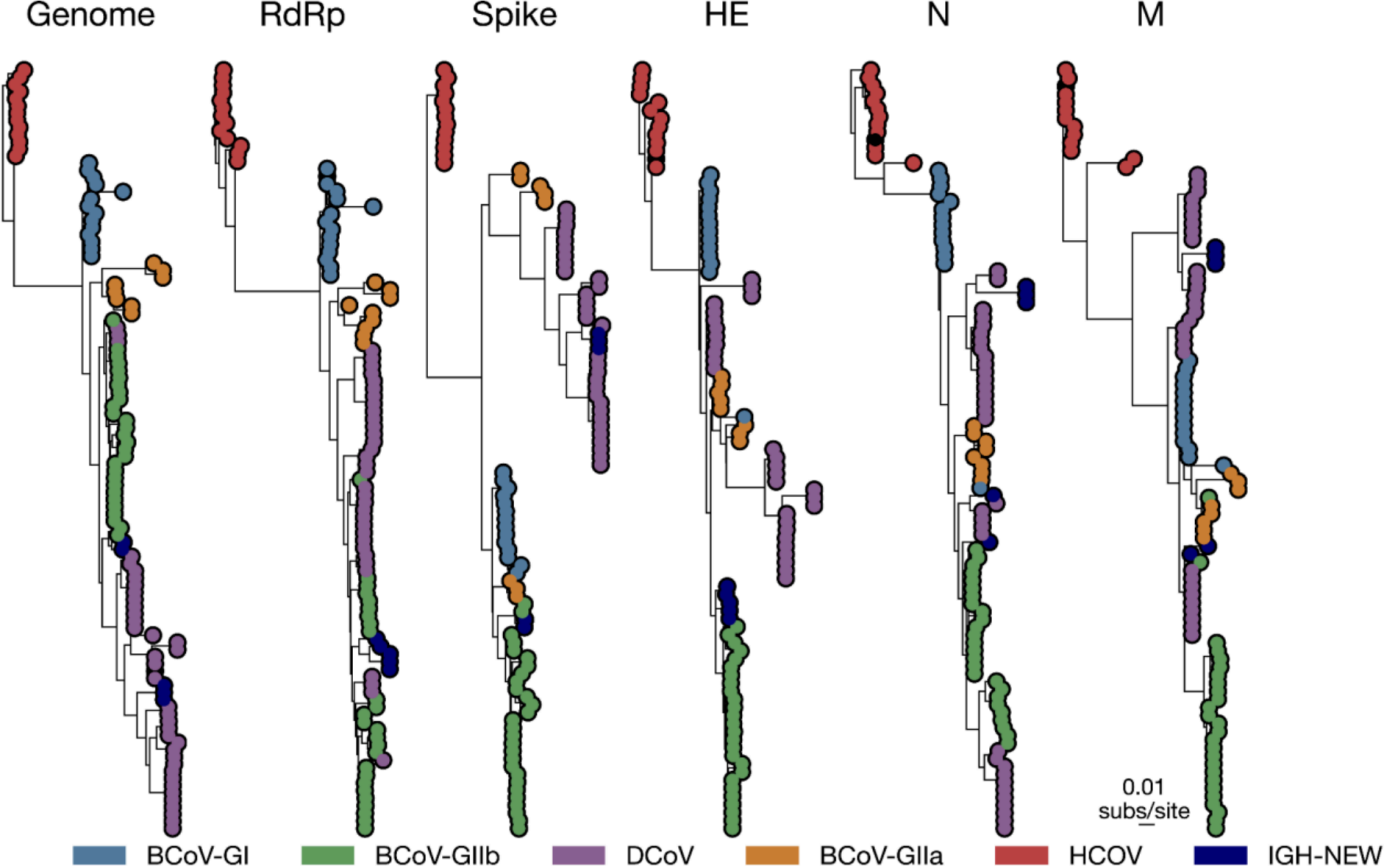
The phylogeny of the Betacoronavirus 1 arranged from: full genome, RdRp (ORF1ab), Spike, Haemaglutinase esterase gene, nucleocapsid, and Membrane genes.

All other coronaviruses identified in this study belong to the *Betacoronavirus* genus, exhibiting >90% aa identity within the RdRp gene region (Figure 4). To resolve Betacoronavirus 1 diversity and evolution in Nigeria, we reconstructed a full-genome phylogeny rooted on human coronavirus OC43^38^. Two distinct β-CoV lineages were identified: three sequences (two goats, one sheep; from Ondo State) formed a well-supported clade (bootstrap >90%) within the *Embecovirus* subgenus, closely related to dromedary camel coronavirus HKU23 strains from North Africa and the UAE^39^, while two sequences (from goats; Ondo and Ebonyi states) formed a sister group to bovine coronavirus (BCoV) genotype 1b strains circulating in Europe^38^ Figure 4, Figure 5). Notably, these two goat sequences exhibited a major deletion between positions 28001–28009 in alignment with bovine enteric and lung coronaviruses, corresponding to the 4.9 kDa protein, though this genomic region is not previously recognized as being under selective pressure^40^.

A hallmark feature of coronaviruses is recombination^41–45^, frequently resulting in alternative tree topologies depending on the analysed genomic region^46,47^. To characterize recombination in the betacoronavirus diversity identified in this study, we reconstructed phylogenies of the RdRp, spike (S), hemagglutinin-esterase (HE), nucleoprotein (N), envelope (E), and membrane (M) genes (Figure 5). The five-sequence RDRP region formed a sister group to the bovine coronavirus (BCoV) that is circulating in Europe. In the spike gene tree the Chinese goat isolates^40^ formed a basal lineage to our sequences and HKU23-like viruses, suggesting a distinct goat/sheep-associated lineage. Conversely, in the HE gene tree, all five study sequences are grouped as a sister clade to European BCoV genotype 1b, distinct from African HKU23 strains. Further incongruence was observed in the N and M gene trees, where three sequences clustered with HKU23-like viruses, and two bovine-like sequences grouped separately with European BCoV genotype 1b, indicating incongruent gene histories which may be explained by recombination (Figure 5).

Recombination breakpoints were further investigated using GARD^48^ and RDP4^49^. GARD identified five breakpoints (ΔAICc = −4,998.1 versus no-recombination model), segmenting the alignment into six phylogenetically distinct regions (Figure 5C). Prominent breakpoints occurred near the ORF1a/1b junction (~6.1–6.6 kb) and within the S/HE region (~21.5–23.1 kb). RDP4 supported these findings, identifying hotspots at the ORF1a/1b junction and S/HE region with multiple recombination detection algorithms (supported by RDP, GENECONV, BootScan, MaxChi, Chimaera, SiScan, and 3Seq). These analyses collectively indicate that the Nigerian *Betacoronavirus* 1 population has undergone extensive recombination between HKU23-like and bovine coronavirus lineages, which explains the conflicting phylogenetic placements of sequences across genomic regions. Such recombination events underscore the dynamic evolution at the livestock interface. Given previous reports of caprine coronaviruses related to HKU23 causing enteric disease in goats and calves^40^, further studies are needed to determine if Nigerian strains exhibit similar tissue tropism or pathogenicity.

Other novel viruses identified belonged to the *Nairoviridae* family. A novel nairovirus sharing <80% aa identity to known *Orthonairovirus thiaforaense* RdRp was identified from a shrew sample *Suncrus*, forming a distinct sister lineage to previously established lineages from Senegal (Figure 4). Notably, both our shrew-derived sequences and those from Senegal^50^ suggest shrews as likely reservoirs for this viral lineage.

Within the *Picornaviridae* family, several novel viruses exhibiting aa identities ranging from 20–80% to their closest match were identified, predominantly clustering among the unclassified *Picornaviridae*: Riboviria, Kage, and Ousland Picornavirales previously described from animal samples deposited on NCBI (Figure 4). Certain picornaviruses demonstrated clear host-specific clustering patterns. Sequences exhibiting >90% aa identity to *Aichivirus* were host-specific, with goat sequences clustering with *Aichivirus A* from ovine samples, and pig sequences aligning with *Aichivirus C* previously identified from pigs. Similarly, *Enterovirus G* isolates from pigs clustered with known pig-derived sequences. Of clinical relevance, several identified picornaviruses belong to high-priority WHO-listed *Enteroviruses* and *Hepatovirus*. We identified *Hepatitis A virus* (HAV) sequences from goats in Ebonyi State, which clustered within genotype 1b alongside Nigerian human-derived sequences. This clustering pattern was consistent at the nucleotide full genome and VP1 segment level, associating closely with isolates previously identified in Lassa fever patients^33^ (Figure 3, Supplementary Figure 5) indicative of occasional spillover into humans from animals within human habitats particularly among children,^51^. Additionally, Echovirus (*Enterovirus B*) isolates clustered with Nigerian human sequences, while Coxsackievirus (*Enterovirus C*) isolates displayed broad dispersal across human sequences from Nigeria and Cameroon. Rhinoviruses, responsible for common colds in humans, clustered predominantly with human-derived isolates from Kenya. All these viruses are transmitted faecal-orally and have been identified as a significant public health threat causing a variety of illnesses ranging from the common cold to aseptic meningitis and extensive morbidity in both humans and animals with significant economic impact globally^52^. Hence the presence of these viruses among animal populations in human dwelling environments suggests the risks of spillover into humans and leads to major outbreaks. On the other hand, we do not have enough data to evaluate the reverse transmission from humans into animals.

In contrast, the dsRNA *Picobirnaviridae* family, exhibiting the broadest host diversity across six of eleven sampled taxa, did not cluster predominantly by host species (Figures 3A, 4, Supplementary Figure 4). Novel picobirnavirus sequences sharing 20–80% amino acid identity demonstrated heterogeneous clustering patterns. Dog isolates clustered closely with diverse mammalian picobirnaviruses, including pig *picobirnavirus sp., lysoka-like picobirnavirus, marmot picobirnavirus, dromedary picobirnavirus*, and human isolates. Goat, pig and sheep isolates grouped with ovine or porcine-associated or camel-associated strains. Notably, rodent-derived picobirnaviruses are primarily grouped with pig-associated strains, although certain rodent isolates are clustered separately. Despite some sequences consistently grouped with hosts such as pigs and goats, host specificity appears less relevant for picobirnaviruses due to their widespread and commensal nature^53^. *Picobirnavirus* have been ubiquitously identified across diverse geographic regions and host ranges, including humans, mammals, birds, and reptiles^35–37^. Although generally non-pathogenic, they have been implicated as opportunistic pathogens causing diarrhoea in immunocompromised individuals^53^, further highlighting the importance of continued surveillance and research into their transmission dynamics and pathogenic potential.

Noroviruses from dog and goat from the two locations were >90% aa identity to known viruses, and they clustered together with noroviruses from humans. *Feline calicivirus* was exclusively found in the cat and grouped with sequences previously reported from cat samples^53^. Similarly, all the viruses identified in the *Sedeoreoviridae* have >95% aa identity, rotaviruses A were mainly clustered together with the viruses from human samples, while *rotavirus F* known to be infecting pigs, also clustered together with porcine rotavirus previously reported^53^. Within *Tobaniviridae*, all sequences were >90% aa identity to the RdRp *Torovirus* previously reported. They were also segregated by hosts, with goat and sheep clustered together with the goat and ovine torovirus. While sequences from pigs clustered together with sequences that have been reported in pigs.

Another important high-priority pathogen of humans identified in this study is the Arenaviridae, mainly in the genus Mammarenaviruses, responsible for haemorrhagic fever outbreaks in Nigeria. All the L segments were above >90% amino acid identity to Lassa virus (LASV) previously reported in humans in Nigeria. They all belong to the lineage II and clustered together with sequences that have previously been linked to human outbreaks in Nigeria including those from the 2018 outbreak and earlier years 33,54,55. Three of the sequences formed a tight cluster with each other and were closely related to the sequences that caused an outbreak in 2012 (Figure 4 supplementary figure 5). The remaining sequences were dispersed across the tree between the 2017/2018 outbreaks in Ondo State and Edo State in Nigeria.

Although the amino acid identity of the S segment ranged between 70–90% they showed similar clustering as observed for the L segment, except two of our new sequences from Mastomys coucha that closely grouped with an isolate from Mastomys natalensis from Ondo State in 2015. We sampled 46 rodents in Ebonyi State, including from homes with a history of Lassa fever outbreaks, but found no Lassa virus reads. This absence aligns with previous observations, suggesting geographic or ecological variability in Lassa virus reservoir distribution within Nigeria that might require intense sampling to uncover 29,55.

Within the *Paramyxoviridae* family, we identified *Peste des petits ruminants* virus (PPRV), a member of the genus *morbillivirus*, in goats, sampled from Ebonyi State with aa identity >90% within the RdRp. Both the RdRp gene and the complete genomes revealed that they belong to lineage IV and are closely related to strains previously identified in goats from Plateau State, Nigeria, in 2020^56^. Interestingly, our two sequences were distributed across the plateau outbreak and were not found to be clustered together on the phylogeny, and we did not observe a direct link to the previously reported sequence (figure 4, supplementary figure 5).

DNA viruses within the *Parvoviridae* family also exhibited broad host distribution, being detected in bats (*Eidon helvum*), dogs, pigs, goats, and rodents (genus *Sciurus* and *Mastomys*). Canine parvovirus sequences from dogs and pigs collected in Ondo State clustered closely with previously reported Nigerian isolates, highlighting a significant veterinary concern given their known pathogenicity in dogs and broad host range across humans, cattle, cats, and other ungulates^53,57^. *Dependoparvovirus* sequences from squirrels formed distinct clusters, while *Protoparvovirus* sequences showed clear host-specific clustering patterns. Dog-derived sequences grouped with previously reported dog isolates, and goat-derived sequences clustered with known goat isolates irrespective of geographic origin. Similar host-specific clustering was observed for adeno-associated and boca parvoviruses. Additionally, *Parapox orf* virus of the *Poxviridae*, was identified in goats and pigs and formed distinct lineages that were distantly related to previously reported strains, suggesting novel diversity within this virus family.

## Potential cross-transmission across detected viral families

**Figure 6:**
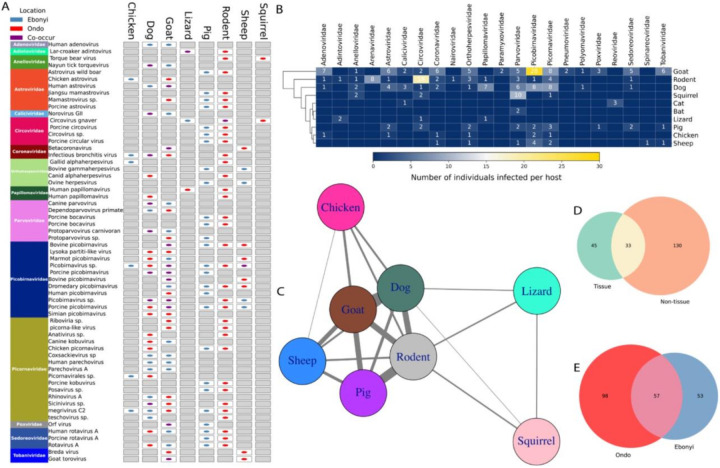
Cross-species virus transmission and host-virus network structure across sampled animals. **a,** Dot plot illustrating the presence and geographical distribution (Ebonyi in blue, Ondo in red, and co-occurrence in purple) of various viral families across different animal hosts. Each row represents a specific virus taxon, grouped by viral family, and columns represent host species sampled. **b,** Heatmap depicting the number of individual animals infected per host species across identified viral families. Darker colours indicate fewer infections, while lighter colours (yellow) represent higher numbers of infected individuals, emphasizing infection prevalence and host-specific patterns. **c,** Network analysis visualizing interspecies viral sharing among sampled hosts. Nodes represent host species, and connecting lines illustrate the sharing of viral taxa between species. Thicker lines indicate stronger viral connections, highlighting significant cross-species transmission routes, especially among rodents, goats, pigs, and sheep. **d,** Venn diagram comparing viral species richness between tissue and non-tissue samples, illustrating distinct and overlapping viral taxa across different sample types. **e,** Venn diagram depicting the overlap in viral species richness between Ondo and Ebonyi sampling locations, demonstrating both shared and unique viral diversity between the two geographic regions.

Phylogenetic analysis revealed that most viral sequences are clustered primarily by host species, with closely related viruses from similar hosts grouping irrespective of geographic origin except those grouping together with known human viruses (Figure 4, Supplementary Figures 4–5). Hence, we sought to understand potential cross-species transmission among all the animals sampled across ecological locations. We identified 58 viruses detected in multiple host species, spanning 15 viral families, including *Picornaviridae, Astroviridae, Parvoviridae*, and *Coronaviridae* (Figure 5A). Among these, 41 viruses were detected in two host species, 9 in three, 5 in four, 2 in five, and 1 virus (*Picobirnavirus* sp., *Picobirnaviridae*) was found in six host species (Figure 6A). This multi-host detection pattern suggests frequent viral exposure or sharing among ecologically connected species, with viral richness per host driven by diverse viral lineages rather than single dominant infections (Figure 6B).

Among viruses shared between two hosts, distinct patterns emerged. *Anativirus sp. (Picornaviridae)* was restricted to dogs and rodents in Ondo, while *Anelloviridae sp*. co-occurred in *Mastomys* and *Sciurus* in Ondo. *Astrovirus wild boar (Astroviridae) Gammacoronavirus (Rodent associated infectious bronchitis)* and *Betacoronavirus (Coronaviridae)* exhibited cross-location detection, appearing in *Mastomys*, dogs, goat and goats/sheep, respectively, in both Ebonyi and Ondo states. Conversely, *Bovine gammaherpesvirus* (Orthoherpesviridae) was detected in pigs and sheep only in Ebonyi, suggesting that multi-host viruses may still show geographic restriction, but this could be as a result of heterogenous samples of these animals in both locations.

Viruses with broader host ranges displayed more complex distributions. Of the nine viruses infecting three hosts, eight were recovered in both collection sites. *Canine parvovirus (Parvoviridae)* persisted in dogs, goats, and sheep across Ebonyi and Ondo, whereas *Human papillomavirus (Papillomaviridae)* from *Mastomys coucha* appeared solely in Ondo. The remaining seven three-host taxa (including *Chicken picornavirus, Picornavirales sp., Circovirus gnaver, Mamastrovirus sp., Astrovirus wild boar, Marmot picobirnavirus, and Sicinivirus sp*.) similarly bridged both locations, further emphasizing the role of host ecology in shaping cross-species transmission^2^ (Figure1C). Among the five viruses found in four hosts, B*ovine picobirnavirus (Picobirnaviridae)* and *Rotavirus A (Sedoreoviridae)* were consistently detected in goats, pigs, *Mus musculus*, and sheep in both locations. The two viruses with five-host ranges included *Porcine picobirnavirus (Picobirnaviridae)* (dogs, goats, pigs, *Mastomys*, sheep) and *megrivirus C2 (Picornaviridae)* (chickens, dogs, goats, pigs, rodents). Remarkably, *Picobirnavirus sp. (Picobirnaviridae)* had the widest host range, appearing in six species (chickens, dogs, goats, pigs, *Mastomys, Mus*, sheep) across both regions, suggesting either exceptional cross-species transmission potential or high adaptability within these populations.

Network analysis highlighted key inter-hosts viral sharing patterns (Figure 6C). Domestic animals formed a highly interconnected core, with goats exhibiting the most extensive viral associations. This study found that the most frequent viral connections were observed between goats and rodents, mainly involving *Picobirnaviruses* and *Rotaviruses*. Goat-sheep interactions followed, mediated by *Astroviruses* and *Picobirnaviruses*. Agamid Lizards and *Sciurus* were found to share fewer viruses, occupying more peripheral roles, while chickens had moderate connectivity through *Astroviruses* and *Picornaviruses*. Viruses with a broader host range were distributed across both Ebonyi and Ondo regions, whereas those found in only one location were generally limited to fewer hosts. Notably, seven out of 41 viruses identified in two host species demonstrated co-occurrence, being present either in both regions or across multiple hosts within a location.

## Ecological drivers of virus distribution and abundance

The heterogeneous distribution of viruses across hosts and geographic locations prompted a testable hypothesis regarding factors influencing virome diversity and abundance. To address this, we compared virome richness (number of viral taxa) and abundance (viral read counts) across sampled hosts in Ondo and Ebonyi. We used the vertebrate associated viruses and evaluated six predictor variables: host species, host sample size, geographic location (Ondo vs. Ebonyi), sample type (tissue vs. non-tissue), host domestication status (wild, domesticated, peri-domesticated), and host geographic distribution (hosts co-sampled in both locations vs. location-specific). Sample size heterogeneity across locations was explicitly incorporated into models to account for sampling bias. We also added diet into the model by grouping plant eating vertebrates as herbivores and those that eat every other thing other than plant as others.

**Figure 7:**
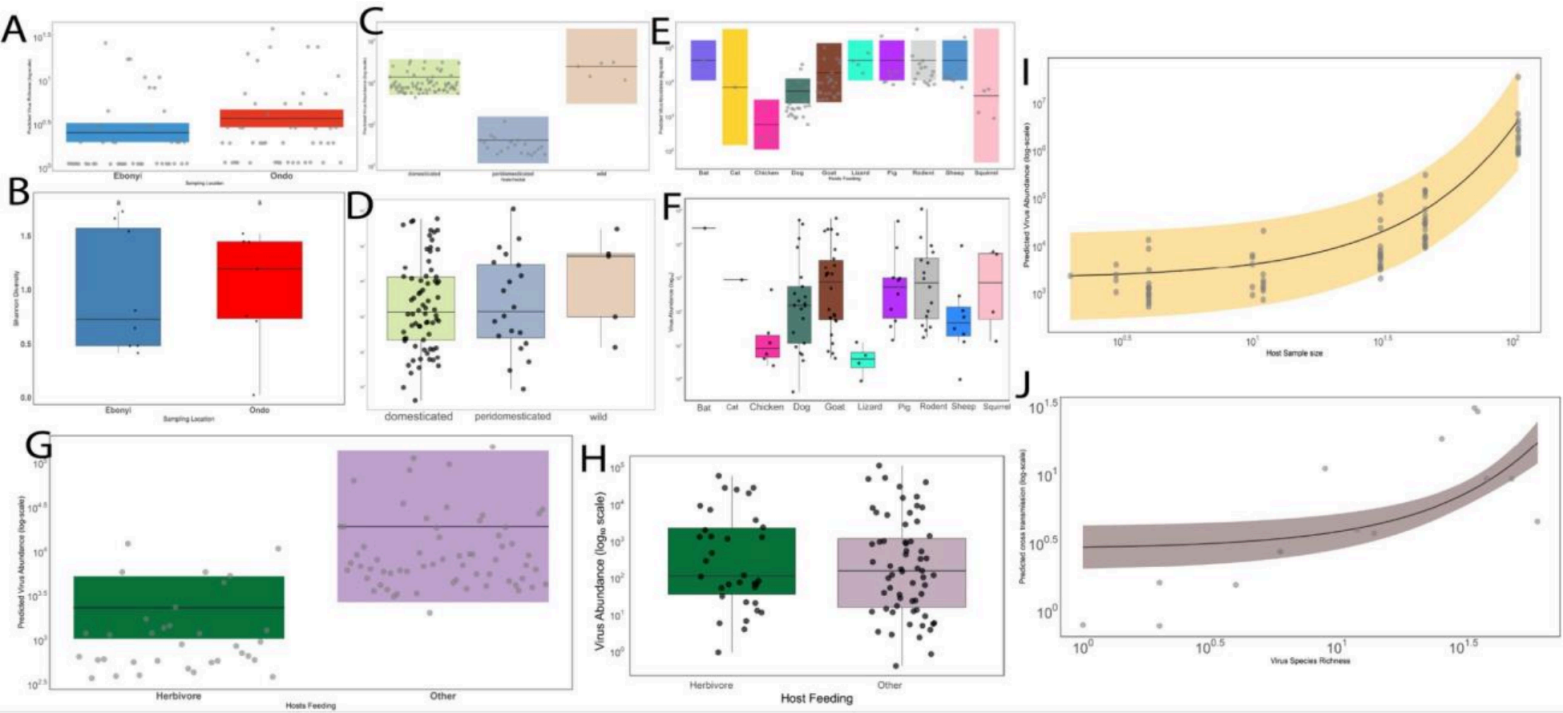
Ecological drivers of virus distribution and abundance. **a,b,** Boxplots comparing predicted viral species richness and Shannon diversity indices between Ebonyi and Ondo sampling locations. Each dot represents individual host samples. **c,d,**Boxplots illustrate predicted viral abundance and shannon index for viral abundance habitat types (domesticated, peri-domesticated, wild), highlighting variations in viral diversity across ecological niches. **e,f,** Boxplots displaying the predicted viral abundance (log-scale) across different host species. Rodents exhibited the highest average viral abundance, followed by goats, pigs, and dogs, while lizards had the lowest abundance. Each boxplot shows median, interquartile range, and outliers. **g,h,** Boxplots comparing predicted viral abundance between herbivores (goats, sheep) and other feeding groups (omnivores, insectivores, carnivores), showing significantly lower viral loads in herbivores. **i,** Scatterplot with regression curve depicting the significant positive correlation between host sample size and predicted viral abundance, emphasizing increased pathogen detection with larger sample sizes. Shaded areas indicate 95% confidence intervals. **j,** Scatterplot with a regression curve illustrating a strong positive correlation between viral species richness and the predicted number of cross-species transmitted viruses, supporting the hypothesis that increased viral diversity heightens spillover risk. The shaded area represents the 95% confidence interval.

Our best-fit models, selected using the Akaike Information Criterion (AIC), explained 13.14% of the variance in viral richness and 21.1% of the deviance in viral abundance (Table 1). While geographic location appeared influential for viral richness, it did not reach statistical significance (Kruskal-Wallis, p = 0.053) (Fig. 7A–B). Location explained 7.04% of the variance in richness, partly attributable to sample size differences and host exclusivity to specific locations. Host habitat explained 6.08% of the variance, with domesticated (Kruskal-Wallis, χ^2^ = 6.48, p = 0.0109) and peri-domesticated animals exhibiting higher viral richness than wild animals. This is one of the limitations of this study given the fewer wildlife sampled in this study are mainly from Ondo State (Fig. 7C–D). This pattern may reflect increased viral sharing opportunities among domesticated animals confined to shared locations, compared to wild hosts with broader ranges. Sampling effort in Ondo State also significantly influenced richness (χ^2^ = 12.81, p = 0.0252).

To address potential non-linearities and unmeasured heterogeneity, we then fitted a Generalized Additive Model (GAM) incorporating the same linear predictors plus random-intercept smooths for host species and collection sites. This GAM explained 14.6% of the deviance in richness. Within this model, sample size positively predicted richness (p = 0.002), and collection site exhibited a strong random effect (p = 3.2×10^−5^). Tissue samples yielded significantly higher richness than non-tissue samples (p = 0.025), and wild hosts had significantly lower richness than domesticated species (p = 0.0013) this could be as a result of sampling bias. Re-analysis using only non-tissue samples revealed consistent factor influences, with sample size per location remaining significant. Samples from Ondo State exhibited higher richness than those from Ebonyi (p < 0.001), and location-specific hosts harboured significantly more viral taxa than widely distributed hosts (p < 0.001). An interaction term between host distribution and sampling location did not improve model fit, indicating additive rather than synergistic effects of these fixed factors.

Viral abundance differed significantly among host species (Kruskal-Wallis, χ^2^ = 18.36, df = 9, p = 0.006). Cats exhibited exceptionally low abundance (p = 0.0013), the agamid lizards the lowest mean abundance (5 RPM), and egrets also showed low abundance (β = −9.11 ± 1.49; p < 1 × 10^−9^). Goats displayed moderately elevated loads (β = +1.44 ± 0.57; p = 0.0116). Rodents exhibited the highest mean viral abundance (9,542 RPM), followed by goats (6,981 RPM), pigs (6,002 RPM), and dogs (5,625 RPM) (Figure 7E–F). Hosts with high viral loads usually serve as a potential reservoirs or incidental hosts for viral spillover^2,29,37^, the relative abundance of the viruses within these animals that interphase between wildlife, domesticated and human dwelling likely pose a zoonotic risk. Host habitat strongly affected abundance (Kruskal–Wallis H = 6.93, df = 2, p = 0.031): peri-domesticated (**37.6** RPM) hosts harbored significantly fewer viral reads than domesticated (**190** RPM) animals (β = −41.54 ± 13.34; p = 0.0018), and wild (wild **37.7** RPM) hosts carried lower loads than domesticated hosts (β = −8.30 ± 2.35; p = 0.0004). This may relate to sample type, as tissue samples generally showed higher abundance, potentially reflecting active infection rather than environmental exposure. The model suggested that diet influenced viral abundance (p < 0.001), (Figure 7G–H), however, diet showed no overall association with RPM (Kruskal–Wallis H = **5.19**, df = **4**, p = **0.268**; Fig. 7G–H). This could be due to reduced dietary pathogen exposure and given that plant viruses were not included in this analysis.

A complementary GAM for abundance, incorporating random-intercept smooths for host taxa and collection site, explained 22.7% of the deviance. Tissue sampling remained a strong predictor (β = 4.14 ± 0.76; p = 6.1 × 10^−8^). The host taxa smooth was highly significant (edf = 7.35; χ^2^ = 94.1; p < 2 × 10^−16^), indicating substantial inter-taxa variation, and the collection-site smooth also contributed significantly (edf = 0.91; χ^2^ = 39.4; p = 0.0011). Re-analysis stratified by sample type (using the same predictors) confirmed significant contributions of sample type (with rectal swabs showing higher abundance than oral swabs, suggesting differential shedding), location, and host species (all p < 0.001, likelihood-ratio tests). Collectively, sample type and host taxa were the principal determinants of viral abundance, with habitat, diet, and sampling effort playing secondary roles. Sample size (Figure 7I) was a strong predictor for both richness and abundance, underscoring its importance for accurate prevalence estimation.

A model predicting the presence of viruses found in multiple hosts (indicating cross-species transmission potential) explained 72.1% of the deviance (Figure 7H). While this result supports the hypothesis that higher overall viral diversity could increase spillover risk^2,37,58^; however, this will require more extensive sampling and targeted study design to answer this question in the context of Nigeria.

In summary, our findings demonstrate that viral diversity and abundance in vertebrates are shaped by complex interactions among sampling effort, geographic location, sample type, host species, habitat, and diet. This highlights the critical importance of targeted surveillance at human-animal interfaces to effectively predict and mitigate zoonotic spillover events.

## Discussion

We performed the surveillance of human dwelling habitat mammals and wildlife to understand the diversity, ecology, and evolutionary relationship of viruses in domesticated, peri-domesticated, and wild animals across two Nigerian states. Large viral families were identified from all mammals sampled apart, from cattle egret, where we did not identify any viruses. The richness and abundance of pathogens identified in our study were significantly greater than previously reported^29,33^, highlighting a notably high number of novel viruses. Viruses were detected across all animal taxa sampled, with goat, rodents (*Mastomys, Rattus, Mus*), dog and pig exhibiting the highest viral diversity and abundance, indicating their potential role as critical reservoirs or incidental hosts thereby forming a spillover risk.

We identified viruses in multiple animals sampled across different ecological niches, some at high prevalence, indicative of relatively frequent cross-species virus transmission. Notably, we did not identify SARS-CoV-2, but we identified some avian-like coronaviruses in rodents, goats, dogs, and chickens that are closely related to the infectious bronchitis virus (IBV). Gammacoronaviruses are mainly found in birds^58^. The occurrence of avian-origin coronaviruses, traditionally associated solely with birds, in diverse mammalian hosts may indicate historic or recent cross-species transmission events. This is not surprising given that viruses from birds occasionally jump into mammalian hosts, patterns observed with influenza viruses infecting cattle and other ungulates^59^. However, the presence of these viruses in novel mammalian hosts might not represent recent host-switching events but rather undersampling, especially in regions previously undersampled like Nigeria^2^. Expanded surveillance using metagenomic approaches could enhance detection of these viruses in non-traditional hosts, highlighting the flexibility and fluidity of viral host range^20^. Further investigations are critical to assess the zoonotic potential of these viruses among human populations. We also showed some dynamic evolutionary processes, particularly evident in the *Betacoronavirus* genus. Notably, recombination between camel and bovine-associated coronavirus lineages indicates significant genetic reshuffling, potentially driven by livestock interactions. This finding highlights substantial zoonotic and agricultural risks, particularly with coronavirus lineages identified in goats and sheep.

Lassa virus remains a major public-health threat in Nigeria, with recurrent haemorrhagic fever outbreaks across West Africa^29,33,55^. We also detected Thiafora orthonairovirus (*Orthonairovirus thiaforaense*) in shrews, previously reported in Senegal^35,50^. Although Thiafora has not been linked to human disease, related *orthonairoviruses* such as Crimean–Congo haemorrhagic fever virus cause severe illness (^35,50^). The zoonotic potential of *Arenaviridae* and *Nairoviridae* is amplified by the peridomestic ecology of *Mastomys* and *Suncus*, which interface with wild life and frequently occupy human settings. The zoonotic potential of members of *Arenaviridae* alongside other members of the *Nairoviridae* family, raises concerns for public health as the reservoir hosts *Mastomys* and *Suncus* are frequently found within the human habitats posing a risk of spillover. For instance, anthropogenic change: conflict, climate-driven habitat disruption, live-animal markets, agricultural intensification, illicit wildlife trade and encroachment, further increases cross-species contact^9,13,60,61^. Rapid global spread facilitated by population growth and interconnected travel networks could narrow the window for effective containment measures^12–14^. This potential for cross-species transmission emphasizes the importance of ongoing surveillance efforts aimed at understanding the dynamics of viral emergence and the risks posed by zoonotic pathogens.

Enteric viruses impose a substantial global burden, particularly in children^62^. In animals living near people, we detected *echoviruses, coxsackieviruses, adenoviruses*, and *noro-/rotaviruses* which are agents linked to meningitis, myocarditis, and acute gastroenteritis—underscoring a clear public-health risk^62,63^. The sequences show broad genetic diversity yet share ≥90% amino-acid identity with human clinical isolates, indicating strong potential for cross-species transmission^33,53^. Such hosts may sustain persistent faecal–oral shedding, contaminating shared environments and facilitating transmission to cohabiting communities^63^. The reciprocal risk, human-to-animal transmission, remains largely unquantified in Nigeria. Sustained molecular surveillance, alongside targeted interventions (e.g., vaccination and WASH/water-safety measures), is warranted to track viral evolution and mitigate spillover.

There were several viruses of veterinary importance *Paramyxoviridae*, and *Parvoviridae*. *Peste des petits ruminants* (PPR) is a contagious viral disease of sheep and goats with high morbidity reaching 80% to 100% and mortality rates 50% to 90%, which is a major barrier to sustainable small ruminant production and dependent livelihoods and economies^4^. Consequently, PPRV is the only livestock disease currently targeted by a Global Eradication Programme (GEP), which aims to rid the world of PPR by 2030 through vaccination of livestock and thereby contribute to achieving the Sustainable Development Goals. Despite ongoing global eradication efforts spearheaded by the World Organisation for Animal Health (WOAH) and the Food and Agriculture Organization of the United Nations (FAO), PPRV continues to severely impact the economy and food security. Our findings suggest that PPRV remains endemic in Nigeria, underscoring the need for extensive and systematic sampling to better understand viral dynamics and inform effective control and elimination strategies. Parvorviridae is a highly contagious veterinary virus.

Our multi-factorial ecological analysis showed that host speciesare the principal determinant of viral richness, a factor that ranks higher than the effects of sampling size and location. This primary association is further modulated by the hosťs feeding ecology and habitat, illustrating the complex, layered determinants of viral community structure. We also showed that viral richness is positively correlated with sample size, which reinforces the imperative for expanded and strategically designed surveillance to capture a true representation of viral diversity.

Several metagenomic studies have justifiably biased towards zoonotic spillover from animals to humans^1,2,4^, such events represent only a fraction of the total transmission landscape^13,14^. The vast majority of cross-species events likely occur between wildlife and domestic animal populations before getting into humans, creating a dynamic evolutionary ecosystem for novel pathogens emergence, particularly in low-resource human-animal interface^9,21,22^. Our best fit model for the number of viruses found in more than one hosts as a proxy for cross-species transmission showed a strong relationship between total viral richness and cross-species transmission potential, aligning with the hypothesis that higher pathogen diversity increases spillover risks^37^. However, due to the limitation of differential sampling across hosts and location and not sampling multiple sample types for each individual animal sampled in our dataset, we were unable to resolve which specific host taxa pose the greatest zoonotic threat.

Collectively, this study provides an empirical framework for assessing viral spillover risk at the human-animal interface in Nigeria. It shifts the paradigm for surveillance away from a singular focus on historically recognized reservoirs, such as bats, toward a more quantitative, risk-based approach at the human-animal interface. We establish that assessing the total viral richness and abundance among cohabiting animal species with humans is a more effective strategy for identifying transmission hotspots. These insights are fundamental for designing targeted, next-generation early-warning systems and building robust pandemic preparedness in regions of intense ecological overlap.

## Methods

### Ethical Approval

The animal study protocol was approved by the review board of the National Veterinary Research Institute (NVRI), Animal Ethics Committee (AEC) (protocol code AEC/02/143/23).

### Study Design and Sample Collection

A cross-sectional ecology network study was conducted to assess zoonotic pathogens across selected agricultural gradient areas in Ondo and Ebonyi States, Nigeria. During this pilot phase of the project, wild, domestic and peridomestic animals in direct or indirect contact with humans were sampled for viral pathogens detection using metagenomic sequencing. In Ondo State, sampling was done in three communities located in Ifon, Ogbese, and Owo, while in Ebonyi State, animals were sampled in two communities, Nwezenyi and Amana. Sampling took place between December 2023 and March 2024.

A total of 240 domestic and small-bodied wild animals residing in or near human habitations were sampled. These included sheep (10), goats (47), pigs (4), dogs (33), cats (2), chickens (4), lizards (3), cattle egrets (10), squirrels (10), bats (10), and small rodents and shrews (107). Small rodents and shrews were captured using Sherman live-capture traps (H.B. Sherman Traps, Tallahassee, FL, USA) as outlined by Happi *et al*. (2022). Bats were collected from trees and house ceilings, while cattle egrets, squirrels, and African giant rats were captured on farms where agricultural activities occurred, utilising recommended trapping protocols. Captured animals were sedated with chloroform. Blood, oral, and rectal/cloacal swabs were collected where possible. For small rodents and shrews, blood and tissue samples (liver, spleen, lungs) were collected via sterile necropsy.

Blood was collected in EDTA tubes, and swabs or tissues in sterile 2ml snap vials with DNA/RNA shield^™^ (ZYMO RESEARCH). Plasma was processed from blood in the lab. All samples were duplicated and transported to the laboratory at −20°C and −80°C using cold chains. Trained veterinarians performed the collection, following humane and hygienic procedures.

### Nucleic Acid Extraction and Sequencing

Total nucleic acid was extracted from 100 μl of plasma, swabs, and homogenized tissues using the AviPure Rx^™^ kit and AviPure AX96^™^ instrument as per manufacturer guidelines. Libraries were prepared following Kapoor *et al*., 2024 and Twist Biosciences protocols, then pooled for liquid hybridization capture with VirCapSeq-VERT probes and Twist Fast Hybridization Reagents. Enriched pools were quantified, normalized, and sequenced as paired ends on an Illumina NextSeq2000 using a P3 (300 cycle) cartridge.

### Bioinformatics Pipelines

#### Read Filtering and Quality Control

Raw sequencing reads underwent rigorous quality control filtering before assembly and analysis (see Supplementary Figure 1 for read retention at each step). The following sequential steps were applied to each dataset to obtain high-quality, reads: Adapter Trimming and Quality Filtering: Illumina adapters and low-quality bases were removed using *Trimmomatic* v0.39 We used a sliding window approach (4-base window, cutting when average Phred quality <20) and trimmed leading/trailing bases with quality <20. Reads shorter than 50 bp after trimming were discarded.

Low-Complexity Filtering: We filtered out low-complexity reads (e.g., homopolymer-rich or simple repeats) using *PriceSeqFilter* v1.2 (Ruby *et al*., 2013). Reads with complexity below default thresholds were removed to avoid spurious alignments and assembly artifacts. This step eliminated reads consisting mostly of single nucleotide repeats or low information content.

We utilized two complementary pipelines: Scylla (v1.2.0_patch) and IDseq (v3.2) for broad pathogen detection and taxonomic classification. Scylla (artic-network) is a Nextflow-based single-sample pipeline that performs adapter trimming and quality filtering (using *fastp* by default) followed by taxonomic read classification with Kraken2 using the comprehensive PlusPF database^64^. This PlusPF database augments the standard Kraken2 library with additional plasmid and viral sequences, improving sensitivity for pathogen detection^64^. We ran Scylla on each dataset to rapidly bin reads by taxa and identify candidate viral reads. Scylla maps all reads to human reads to remove any possible human reads as contamination that likely occur during the process of sample preparations and then performs a *de novo* assembly on each individual sample using megahit.

## Assembly and Taxonomic Assignment

To validate the assemblies and quantify read support, we mapped the quality-filtered reads back to the assembled contigs using *Bowtie2* v2.4.4^65^. Bowtie2 was run in sensitive local mode, and coverage depth for each contig was calculated. Contigs with reads <2 were excluded from further analysis to avoid spurious contigs Supplementary Figure 1.

We then assigned taxonomic identities to the assembled contigs using a combination of nucleotide and protein sequence searches. Each contig was compared against the NCBI nucleotide database (NT, built 15 November 2024) using BLASTn^66^ and against the NCBI non-redundant protein database (NR) using DIAMOND blastx (v2.0.15)^67^.

As described by^68^ taxonomic classification of each contig was determined by an LCA (lowest common ancestor) approach combining the BLASTn and Blastx results. In practice, we took the top hits from each search judging by their evalue and examined their taxonomic lineages. If all top hits were from the same genus or family, the contig was assigned to that taxon. If hits differed (e.g., some viral, some bacterial), we assigned the contig to the most specific lineage common to most hits. Priority was given to viral hits if any were present, given our focus on virus discovery. Contigs with only very generic hits (e.g., “unknown” or repetitive elements) remained unclassified.

### Background subtraction and contamination control

To quantify and remove background nucleic acids introduced by reagents, surfaces, or personnel, we processed eight no-template (water) controls in parallel with the animals sampled. Following Batson *et al*. (2021), read counts for each taxon were converted to an estimate of nucleic-acid mass by normalizing to ERCC spike-in reads (fixed spike-in volume per library). For each taxon tt, the mean contaminant mass was estimated from the water controls, and a detection in any animal sample was flagged as putative contamination when its estimated mass was < 100× the water-control mean for that taxon. By Markov’s inequality Pr(X≥100 E[X])≤0.01Pr(X≥100E[X])≤0.01, this threshold corresponds to a ≤ 1% false-positive rate. All contigs and reads assigned to flagged taxa in that sample were removed before downstream analyses. “Non-host reads assembled into contigs” refers to reads mapping to contigs that remained after host-depletion and decontamination; “non-host reads” additionally includes filtered reads that did not assemble or formed only minimal (e.g., two-read) contigs.

## Viral Genome Curation and Identification

To curate candidate viral contigs and distinguish true viral genomes from false positives, we applied several additional analyses focusing on hallmark viral features. To reduce redundancy and group together sequences representing the same virus, we clustered the viral contigs using CD-HIT-EST^69^ at 90% nucleotide identity and 90% overlap. This clustering grouped nearly identical sequences (often recovered from different samples) into single representative contigs. For each cluster, the longest contig was chosen as the representative “genome” for further analysis. This step prevented over-counting the same virus present in multiple samples and simplified downstream curation.

A key criterion for novel RNA viruses is the presence of an RNA-dependent RNA polymerase (RdRp) gene. We screened all representative contigs from each cluster for RdRp domains. To do this, we used a custom Python script adapted from (^68^ to predict open reading frames (ORFs) on all contigs, and any ORF longer than 300 amino acids was queried for conserved motifs of viral proteins using profile Hidden Markov Models (HMMs) from Pfam. Specifically, we used HMMER v3.1b2^70^ to search each contig (and its six-frame translations) against a custom database of RdRp-associated Pfam models (These were RdRP_1 (PF00680, Picornavirales-like and Nidovirales-like), RdRP_2 (PF00978, Tymovirales-like and Hepe-Virga-like), RdRP_3 (PF00998, Tombusviridae-like and Nodavir- idae-like), RdRP_4 (PF02123, Toti-, Luteo-, and Sobemoviridae-like), RdRP_5 (PF07925, Reoviridae- like), Birna_RdRp (PF04197, Birnaviridae-like), Flavi_NS5 (PF00972, Flaviviridae-like), Mitovir_RNA_- pol (PF05919, Narnaviridae-like), Bunya_RdRp (PF04196, Bunyavirales-like), Arena_RNA_pol (PF06317, Arenaviridae-like), Mononeg_RNA_pol (PF00946, Mononega- and Chuviridae-like), Flu_PB1 (PF00602, Orthomyxoviridae-like). We also downloaded all the virus families curated by the^68,71^ which cover diverse RNA virus families. Contigs with significant hits to an RdRp HMM (E-value < 1e-5) were marked as putative RNA virus genomes.

### Lucaprot

Next, we integrated a deep learning approach to identify highly divergent viruses that might evade detection by conventional homology. We applied LucaProt^71^, an AI model that integrates sequence and structural features of known RdRps to detect remote homologs beyond the reach of HMMs or BLAST. We ran the LucaProt model on all translated contigs (using the authors’ published parameters and threshold scores) to flag any contigs as likely novel RNA virus RdRps. Indeed, LucaProt helped identify several contigs with no significant Pfam hits, yet which were predicted as RdRp by the model – these were manually examined and kept as novel virus candidates if other evidence (e.g., co-occurrence or secondary structure) also supported them.

All contigs that passed these filters (presence of RdRp domain by HMM or LucaProt, or other strong viral protein hits) were curated as viral contigs. We also cross-checked these contigs against known virus databases (blast results) to see if they matched any previously reported viruses or if they were entirely novel. In cases where multiple contigs were found to originate from the same virus (e.g., different segments of a segmented genome or the same genome split across contigs), we performed further analyses as described below.

## Segment Co-occurrence Analysis

For viruses with segmented genomes, no single segment may contain the RdRp, so we implemented a co-occurrence analysis to link segments belonging to the same virus. We leveraged the fact that all genome segments of a given virus should co-occur in the same samples if they truly originate from one virus as previously described by^68,72^. To systematically identify such associations, we looked at each cluster contigs across the 240 samples and checked for co-occurrence frequencies with contigs identified as RdRp either by the pfam homology or lucaprot. Pairs or groups of contigs that were observed together in the same samples at a high frequency were flagged as potential segments of one virus. We set a stringent threshold that contigs must co-occur in ≥80% of the samples in which either is detected to be considered linked. This 80% threshold is in line with previous recommendations for delineating segmented viral genomes in metagenomic data as described by^68^

### Final Viral Classification Criteria

We established a set of final criteria to designate a contig (or a set of contigs in the case of segmented viruses) as a bona fide viral genome in our dataset. A contig was classified as viral if it satisfied *any* of the following: (1) it encodes a recognizable RdRp domain (by HMM scan or LucaProt prediction), indicating an RNA virus; (2) it has a high-confidence taxonomic assignment to a virus from sequence similarity (e.g., BLASTn/Blastx hits to known viral genes with significant identity); or (3) it is linked to other viral contigs through the co-occurrence analysis (as part of a segmented viral genome where at least one segment meets criterion 1 or 2). In practice, most viral contigs in our study met multiple criteria, reinforcing their classification.

Conversely, we imposed strict filters to exclude false positives or ambiguous sequences. Any contig with a top hit as judged by the evlaue and bitscore to the host genome was removed from viral candidate lists, even if it also had weak viral hits, to avoid mistaking host-derived sequences (for example, endogenous viral elements or transposons) as new viruses. We also removed contigs that were too short (<500 bp) or had only very broad taxonomic assignments (e.g., “viral protein”) without any specific hallmark. Contigs matching only bacteriophage or plasmid sequences were set aside from eukaryotic virus analyses, as our focus was on eukaryotic virome; however, notable bacteriophages were still catalogued in the microbiome results. Finally, if a contig had mixed signals (different parts aligning to different taxa), we did not classify it as a single virus but rather marked it as a potential chimera or ambiguous and excluded it from definitive counts.

After these steps, we curated a final list of high-confidence viral genomes, each either represented by a single contig or by a group of segments (with an identifier for each virus). In total, we identified 1676 distinct virus contigs (both known and novel) across the 240 datasets (see Results and Figure 2 for summaries). These include RNA viruses (the majority, all containing RdRp) and a smaller number of DNA viruses identified via nucleotide homology to replicase or DNA pol.

## Microbiome Profiling per Sample

In addition to aggregate community analysis, we profiled the microbiota of each individual sample, with a particular focus on viruses and other microbes of interest. For each sample, we calculated the proportion of non-host reads attributable to each microbial taxon (virus, bacteria, Platyhelminthes, Nematoda, Trypanosomatidae, Apicomplexa). We applied an inclusion threshold of ≥0.1% of non-host reads for reporting a taxon as present in a given sample. In other words, only organisms whose reads comprised 0.1% or more of that sample’s non-host read count were considered part of the core microbiome of the sample. These cutoff filters out extremely low-abundance taxa that might represent contamination or sequencing noise, improving the robustness of per-sample profiles. The threshold was chosen based on read depth and diversity considerations, balancing sensitivity to genuine low-level infections with specificity Using this criterion, we noted that many samples harboured one or more viruses above the 0.1% level. Notably, several RNA viruses (including novel ones identified in this study) exceeded 0.1% of reads in the samples they were found, highlighting them as dominant members of those samples’ microbiomes (Supplementary Figure 4). Eukaryotic microbes were recorded per sample if they crossed the 0.1% threshold.

## Phylogenetic Analysis of Viral Sequences

To contextualize the novel viruses evolutionarily, we performed comprehensive phylogenetic analyses on selected viral genes. We focused primarily on the RdRp protein sequences of RNA viruses, to infer the relationships of our discovered viruses to known lineages. Multiple sequence alignments were constructed using MAFFT v7.475^73^ under default settings (FFT-NS-2 algorithm). Alignments were then trimmed with trimAl v1.4^74^ in automated mode to remove poorly aligned or gap-rich regions, ensuring that only high-confidence columns were used for tree inference. For each virus family or group of interest, we included reference sequences from GenBank (representative RdRp sequences from related viruses) alongside our novel sequences in the alignment, to provide phylogenetic context. We inferred maximum-likelihood phylogenetic trees using IQ-TREE2^75^. For protein alignments, the LG + F amino acid substitution model^76^ with a gamma distribution of rate heterogeneity was selected, as recommended by ModelFinder in IQ-TREE for most RdRp alignments. Node support was assessed with 1000 ultrafast bootstrap replicates (UFBoot2) to ensure robust support values. The resulting trees were visualised using the Python library Baltic.

### Recombination analysis

Recombination breakpoints were investigated using GARD and RDP4. GARD identified five breakpoints (ΔAICc = −4,998.1 versus no-recombination model), segmenting the alignment into six phylogenetically distinct regions (Figure 5C). Prominent breakpoints occurred near the ORF1a/1b junction (~6.1–6.6 kb) and within the S/HE region (~21.5–23.1 kb). RDP4 supported these findings, identifying hotspots at the ORF1a/1b junction and S/HE region with multiple recombination detection algorithms (supported by RDP, GENECONV, BootScan, MaxChi, Chimaera, SiScan, and 3Seq). These analyses collectively indicate that the Nigerian Betacoronavirus 1 population has undergone extensive recombination between HKU23-like and canonical bovine coronavirus lineages, explaining conflicting phylogenetic placements of sequences across genomic regions.

### Ecological factors influencing virome composition and putative viral sharing in vertebrates

To identify ecological determinants of the number of viral taxa (i.e., virome richness), total viral abundance, and the occurrence of putatively cross-species viruses, we fitted independent sets of generalized linear models (GLMs) that enumerated all possible combinations of available covariates. Briefly, for models of virome richness and abundance, candidate predictors comprised host species, host sample size (per species within location), geographic location (Ondo vs. Ebonyi), sample type (tissue vs. non-tissue), host habitat (domesticated, peri-domesticated, wild), host geographic distribution (co-sampled in both locations vs. location-specific), and diet (herbivore vs. other). Richness was modeled using negative-binomial GLMs to correct for over dispersion; abundance models were fit to log1-ptransformed reads-per-million (RPM).

For the model predicting the potential for cross-species detection (binary indicator that a viral taxon was found in ≥2 host species), we used mixed generalized additive models (GAMs) with a binomial link, implemented via mgcv (v1.8–41) with the “REML” method. A virus family-level taxonomic random effect was included to represent relatedness among viruses given that newly identified taxa were often too divergent for robust, genus-level phylogenies. This taxonomic random effect was combined with all possible combinations of additional covariates including mean viral abundance, prevalence, location (detected in one vs. both locations), sample-type, habitat, and diet category of host detections. In addition, we fitted two other independent sets of GAMs that added a random effect of virus family nested within host order or host family, respectively, to test for host-specific virus-family effects (Table 1; Models 5 and 6).

All fitted models were ranked by Akaike Information Criterion (AIC), and the model with the lowest AIC within each set was selected as the best-fit model. Variable importance was quantified by deviance partitioning using drop-one comparisons. Specifically, model deviance explained was calculated as (Dn−Df)/Dn(Dn−Df)/Dn, and the deviance explained by each variable in the full model was calculated as (Di−Df)/Dn(Di−Df)/Dn, where DnDn is the deviance of the intercept-only model, DfDf is the deviance of the full model, and DiDi is the deviance of the submodel excluding the test variable. Partial effects were obtained from the best-fit models by predicting at the most common category for other categorical variables and the median for numeric variables. Sensitivity analyses evaluated specification of sampling effort as a covariate versus offset, restriction to non-tissue samples to assess detection bias, and alternative distributional families for abundance; conclusions were unchanged. Model diagnostics included residual dispersion, influence, and concurvity checks, with basis-dimension reduction when indicated.

## Supplementary Files

This is a list of supplementary files associated with this preprint. Click to download.

- Additionalinformation.docx

Supplementary Tables

Supplementary Tables are not available with this version.

## Figures and Tables

**Table 1:** 

Model for virus richness		
Term	p-value	Deviance Explained (%)
Model 1: virus richness		13.14
location	p < 0.001	7.04
habitat	p <0.001	6.08
Host geographic distribution	p < 0.001	5.04
Sample size Ondo	p < 0.05	5.62
Model 2: virus abundance	–	21.1
Host type	–	13.85
Sample type	0.3170	7.25
Model for Cross-Species		
Model 3: Full Model	–	70.05
Virus richness	p<0.001	

## Data Availability

All code to run the analyses is available in Github https://github.com/Ifeanyi-omah/Project-metagenomics.git and Figshare https://figshare.com/account/home#/projects/263521
